# Dataset of mitochondrial genome variants in oncocytic tumors

**DOI:** 10.1016/j.dib.2018.02.040

**Published:** 2018-02-16

**Authors:** Lihua Lyu, Qiufeng Wang, Shujie Song, Huaibin Zhou, Ming Li, Chen Zhou, Zhiying Jiang, Liyan Li, Jianxin Lyu, Guorong Chen, Yidong Bai

**Affiliations:** a School of Laboratory Medicine and Life Sciences, Key Laboratory of Laboratory Medicine, Ministry of Education, Zhejiang Provincial Key Laboratory of Medical Genetics, Wenzhou Medical University, Wenzhou 325035, Zhejiang, China; b Department of Cellular and Structural Biology, University of Texas Health Science Center at San Antonio, San Antonio, TX 78229, USA; c Department of Pathology, the First Affiliated Hospital of Wenzhou Medical University, Wenzhou 325000, China

## Abstract

This dataset presents the mitochondrial genome variants associated with oncocytic tumors. These data were obtained by Sanger sequencing of the whole mitochondrial genomes of oncocytic tumors and the adjacent normal tissues from 32 patients. The mtDNA variants are identified after compared with the revised Cambridge sequence, excluding those defining haplogroups of our patients. The pathogenic prediction for the novel missense variants found in this study was performed with the Mitimpact 2 program.

**Specifications table**

**Table:** 

Subject area	*Genetics*
More specific subject area	*Oncocytic tumors*
Type of data	*Table, text file*
How data was acquired	*Sanger sequencing of whole mitochondrial genomes*
Data format	*Analyzed*
Experimental factors	*Samples are parafin-bedded tissues*
Experimental features	*The whole mitochondrial genomes from the tumor tissues were sequenced, the variations were identified by comparing sequences with the revised Cambridge sequence (rCRS) (GenBank number*NC_012920*); the predictions for pathogenicity of mtDNA variants were established according to the Mitimpact 2 program.*
Data source location	*Wenzhou, China*
Data accessibility	*The data are available with this article*

**Value of the data**

• The data identified inherited mtDNA variants associated with patients with oncocytic tumors.
• The data showed some identified mtDNA variants could have functional consequences.
• The data might help to detect new genetic predisposition markers for oncocytomas.

## 1 Data

The data were presented as tables, where positions, detail changes (Table 1), and the implications were provided (Table 2).

**Table 1:** MtDNA variants not defining haplogroups of our patients.

Locus	MtDNA variants	Amino acid change	PolyPhen 2 score	Annotation status
TV	m.1623G>A	tRNA valine	–	Novel
RNR2	m.1719G>A	16 s RNA	–	KNP
ND1	m.3398 T>C	Met→Tyr	0.02	cardiomyopathy-associated
ND2	m.4935 A>G	Thr→Ala	0	KNP
	m.5178 C>A	Leu→Met	0.9	Longevity;AMS protection;blood iron metabolism
CO1	m.6221 T>C	N	–	KNP
	m.6267 G>A	Ala→Thr	0.01	Prostate Cancer
	m.6371 C>T	N	–	KNP
	m.6680 T>C	N	–	KNP
CO2	m.8239 C>A	Ile→Met	0.29	Novel
	m.8240 T>A	Phe→Ile	0.99	Novel
	m.8241 T>A	Phe→Tyr	0.98	Novel
CO3	m.9510 T>C	Tyr →His	1	Novel
	m.9698 T>C	N	–	KNP
TR	m.10410 T>C	tRNA arginine	–	KNP
ND4L	m.10490 T>C	N	–	KNP
	m.10530 G>A	Val→Met	0.02	KVP
ND4	m.11531 G>A	Ala→Thr	0.18	KNP
	m.11719 G>A	N	–	KNP
	m.11968 A>T	N	–	KNP
	m.12131 T>C	Ser→Pro	0.05	KNP
	m.12811 T>C	Tyr→His	0.01	Possible LHON factor
	m.13674 T>C	N	–	KNP
	m.13590 G>A	N	–	KNP
	m.13934 C>T	Thr→Met	0.08	KNP
	m.13966 A>G	Thr→Ala	0.01	KNP
ND5	m.14097 C>T	N	–	KNP
ND6	m.14180 T>C	Tyr→Cys	0.96	KNP
CYB	m.14766 C>T	Thr→Ile	0.01	KNP
	m.15746 A>G	Ile→Val	0	breast tumor
D-Loop	m.193 A>G	–	–	KNP
	m.298 C>T	–	–	KNP
	m.310 T>C	–	–	KNP
	m.310 ins C	–	–	Melanoma patients
	m.310 ins CC	–	–	KNP
	m.316 G>C	–	–	KNP
	m.316 GC	–	–	Novel
	m.318 ins CC	–	–	Novel
	m.523 del A	–	–	KNP
	m.524 del C	–	–	KNP
	m.16278 C>T	–	–	KNP
	m.16327 C>T	–	–	KNP
	m.16360 C>T	–	–	KNP
	m.16497 A>G	–	–	KNP
	m.16519 T>C	–	–	KNP
	m.16526 G>A	–	–	KNP

The DNA variants detected both in oncocytic tumors and adjacent normal subjects.

N, no amino acid change; –, Not applicable; KNP, Known sequence nucleotide polymorphism; Annotation status in Mitomap, the mtDNA variants associated with diseases or being a somatic events in other disease summarised in Mitomap database.

**Table 2:** The pathogenic prediction of 4 novel missense mtDNA variants identified in this study.

**Index**	**m.8239 C>A**	**m.8240 T>A**	**m.8241 T>A**	**m.9510 T>C**
**AA position**	I218M	F219I	F219Y	Y102H
**PolyPhen2**	Benign	Probably damaging	Probably damaging	Probably damaging
**SIFT**	Neutral	Neutral	Neutral	Neutral
**FatHmmW**	Neutral	Deleterious	Neutral	Neutral
**PROVEAN**	Neutral	Deleterious	Deleterious	Deleterious
**Mutation Assessor**	Neutral impact	High impact	Medium impact	Medium impact
**EFIN SP**	Neutral	Damaging	Damaging	Damaging
**EFIN HD**	Neutral	Neutral	Neutral	Damaging
**CADD**	Deleterious	Deleterious	Deleterious	Deleterious
**PANTHER**	Disease	Disease	Neutral	Neutral
**PhD-SNP**	Neutral	Disease	Disease	Disease
**SNAP**	Neutral	Disease	Disease	Disease
**Meta-SNP**	Neutral	Disease	Disease	Disease
**CAROL**	Neutral	Deleterious	Deleterious	Deleterious
**Condel**	Neutral	Neutral	Deleterious	Neutral
**COVEC WMV**	Neutral	Deleterious	Deleterious	Deleterious
**MtoolBox**	Neutral	Deleterious	Deleterious	Deleterious
**PolyPhen2 transf**	Medium impact	Low impact	Low impact	Low impact
**SIFT transf**	Medium impact	Medium impact	High impact	Medium impact
**Mutation Assessor transf**	Low impact	High impact	Medium impact	Medium impact
**MutationTaster**	Polymorphism	Polymorphism	Disease causing	Polymorphism

## 2 Experimental design, materials and methods

We collected 32 cases of the parafin-bedded tissues with oncocytic tumor and matched adjacent normal tissues, mtDNA were amplified by PCR using 24 previously reported pairs of mtDNA primers to cover the whole mtDNA genome as our previous work [1]. MtDNA variants were yielded by comparing sequencing results of the complete mitochondrial genome with the revised Cambridge sequence (rCRS) (GenBank number NC_012920). The heteroplasmy were defined if a double peaks of two residues were verified at the same position in the electro-chromatograms. Pathogenic prediction were analyzed using PolyPhen2 (http://genetics.bwh.harvard.edu/pph2/) [2] and MitImpact 2 [3].
