# Supplementary material for: Dataset of mitochondrial genome variants in oncocytic tumors
Source: Data Brief. 2018 Feb 16;17:1149–52. doi: 10.1016/j.dib.2018.02.040 (PMC5988458; doi:10.1016/j.dib.2018.02.040)
Supplement: Supplementary file 1 — Supplementary material [file mmc1.docx]

Conflict of interests

None declared.

Yidong Bai, for all the authors.
